# A case of COVID-19 re-infection in Libya

**DOI:** 10.7196/AJTCCM.2021.v27i2.131

**Published:** 2021-06-23

**Authors:** A Alzedam, A M Bengblya, M J Zeglam, E T Benmassoud, S M Bennji

**Affiliations:** Zliten Isolation Unit, Zliten Medical Center, Zliten, Libya

**Keywords:** COVID-19, reinfection, Libya

## Abstract

Re-infection with SARS-CoV-2 is an active area of research and studying. Here, we present the first documented case of SARS-CoV-2 re-infection in Libya. The patient was a 24-year-old healthy man who initially presented with mild symptoms of generalised fatigue and intermittent episodes of fever for 3 days. During his second episode of COVID-19, he presented with chest tightness and intermittent dry cough. The patient fully recovered from both episodes of COVID-19 without any residual complaints. Since limited cases of SARS-CoV-2 re-infection have been identified, it is probably a rare phenomenon. It is however critical to identify the role of new SARS-CoV-2 variants in the pathogenesis of recurrent COVID-19.

## Background

Since the first case of coronavirus disease 2019 (COVID-19) caused by the severe acute respiratory syndrome coronavirus 2 (SARS-CoV-2) was reported in Wuhan City, China in December 2019, there has been an explosion of publications on disease epidemiology, transmissibility and pathogenesis. Uncertainty however still exists regarding individual immune responses and susceptibility to re-infection. Several reports of SARS-CoV-2 re-infection have been published in China, Italy and Pakistan.^[1,2]^ In view of the limited number of cases reported, re-infection with SARS-CoV-2 is probably a rare phenomenon. Here we report a case of recurrent SARS-CoV-2 infection in a young adult male from Libya.

## Case

A 24-year-old male non-smoker, who had no known chronic illnesses, presented to the fever clinic at Zliten Medical Center in June 2020 complaining of fatigue and intermittent fever for the preceding 3 days. On clinical examination, he was found to be oriented with normal vital signs. Routine blood test results were within normal limits. A chest X-ray was not performed at that stage since the patient had mild flu-like symptoms without any respiratory symptoms. His nasopharyngeal swab result for SARS-CoV-2 polymerase chain reaction (PCR) test was positive on 6 June 2020. Since his symptoms were mild, the patient did not require hospitalisation and was sent home for self-isolation. After a period of 5 days, all of his initial symptoms had improved. A repeat nasopharyngeal swab for SARS-CoV-2 PCR performed 11 days after the initial test was still positive. The SARS-CoV-2 PCR test was performed again 21 days after the initial positive result and was found to be negative. A confirmatory nasopharyngeal SARS-CoV-2 PCR test was conducted 24 hours later and the result remained negative. The patient was therefore allowed to de-isolate and continue normal activities. On 10 August 2020, the patient was again referred to our fever clinic with complaints of mild chest tightness and a dry cough. A nasopharyngeal swab for SARS-CoV-2 PCR performed at that stage was positive, confirming a diagnosis of COVID-19 re-infection. The severity of his symptoms during his second episode of COVID-19 were however milder than the first episode and subsided after a few days. The patient was subsequently declared non-infectious 13 days after a period of self-isolation as per the World Health Organization (WHO) criteria for recovery.

## Discussion

According to the WHO, there is currently insufficient evidence regarding the effectiveness of SARS-CoV-2 antibody-mediated immunity to protect against recurrent episodes of COVID-19.^[3]^ A previous case was reported of a 33-year-old man from Hong Kong who first tested positive for COVID-19 in March 2020 after he developed symptoms of coughing, sore throat, fever and headache. The patient fully recovered from COVID-19 and this was confirmed by negative SARS-CoV-2 PCR analysis. In mid-August of the same year, the patient tested positive for SARS-CoV-2 by PCR again. On assessing him for antibodies against SARS-CoV-2, it was noted that he initially did not have antibodies after his first bout of COVID-19, whereas he did develop antibodies with his subsequent re-infection with SARS-CoV-2.^[4]^ Additionally, we are aware of at least three other cases of recurrent COVID-19 in Zliten city (unpublished).

It has been assumed that people would not become vulnerable to COVID-19 again after recovering from an initial episode, based on how the immune system typically responds to other respiratory viruses, including other coronaviruses. It is possible that only a minority of patients will be re-infected with SARS-CoV-2 and develop recurrent COVID-19. Of importance however are the implications that SARS-CoV-2 re-infection has for virus transmission, vaccine efficacy and controlling the pandemic in general. It is also very important to know what the role of SARS-CoV-2 variants in the pathogenesis of recurrent COVID-19 is. The case reported here contributes to the global literature on SARS-CoV-2 re-infection and highlights some of the unanswered questions regarding the pathogenesis and impact of recurrent COVID-19.
